# Trend and Associated Factors for Late and Advanced HIV Diagnoses in 2011–2022 in Melbourne, Australia

**DOI:** 10.1002/jmv.70430

**Published:** 2025-06-04

**Authors:** Warittha Tieosapjaroen, Arron Tran, Marcus Maisano, Cham‐mill Kim, Christopher K. Fairley, Eric P. F. Chow, Lei Zhang, Tiffany R. Phillips, Melanie Bissessor, Jason J. Ong

**Affiliations:** ^1^ School of Translational Medicine, Faculty of Medicine, Nursing and Health Sciences Monash University Melbourne Australia; ^2^ Melbourne Sexual Health Centre, Alfred Health Melbourne Australia; ^3^ Melbourne Medical School The University of Melbourne, Faculty of Medicine Dentistry and Health Sciences Melbourne Australia; ^4^ Centre for Epidemiology and Biostatistics, Melbourne School of Population and Global Health The University of Melbourne Melbourne Australia; ^5^ Faculty of Infectious and Tropical Diseases London School of Hygiene and Tropical Medicine London UK

**Keywords:** advanced HIV diagnosis, AIDS, HIV, HIV diagnoses, HIV testing, late HIV dianogsis

## Abstract

Late‐ and advanced HIV diagnoses remain significant challenges worldwide. Understanding the factors associated with and testing practice among those with late (CD4 < 350 cells/µL) and advanced diagnoses (CD4 < 200 cells/µL) is essential to diminish AIDS‐related morbidity and mortality. This retrospective study manually reviewed e‐medical records of new HIV diagnoses between 2011 and 2022 at Melbourne Sexual Health Centre, Australia. Among 606 new diagnoses, 25% (152/606) were late, 11% (65/606) were advanced, and 94% (568/606) were men who have sex with men. Among 352 overseas‐born individuals, late or advanced diagnoses increased from 22% (4/18) in 2011 to 56% (5/9) in 2022. No significant change was observed among Australian‐born individuals. Late diagnoses were associated with being born in Latin America or in East Asia and Pacific, having no prior sexually transmitted infection (STI) diagnosis in their lifetime, and no drug use in their lifetime. Advanced diagnoses were associated with being born in East Asia and the Pacific, having no prior STI, older age, Medicare‐ineligibility, and unknown condom use history in the last year. Key barriers to earlier testing included LGBT‐related stigma (*n* = 41) and recent arrival (< 5 years) in Australia (*n* = 41). The primary risk for contracting HIV was condomless anal sex (*n* = 80). To conclude, one in three new HIV diagnoses was late or advanced, with increased risk among overseas‐born, Medicare‐ineligible or those with perceived low risk. Tailored campaigns for these underserved populations are urgently needed to promote timely HIV testing.

## 1 Introduction

The Joint United Nations Programme on HIV/AIDS (UNAIDS) aimed to end the AIDS epidemic by 2030 [1]. While significant strides in HIV care have been made in the past decades to achieve this goal, significant challenges remain. For example, in Australia, high levels of testing, pre‐exposure prophylaxis (PrEP) coverage and antiretroviral therapy (ART) use among people living with HIV (PLHIV) have significantly reduced HIV incidence [2, 3]. However, among 722 new HIV diagnoses in 2023, 37% were classified as late diagnoses (defined as CD4 count < 350 cells/µL) [4].

Despite the UNAIDS's Fast Track Target of ensuring that 95% of PLHIV are aware of their HIV status by 2025, only nine countries have claimed to achieve this target in 2023 [1]. Additionally, in countries with a high proportion of PLHIV aware of their HIV status, disparities exist among underserved populations. For example, 93% of PLHIV in Australia are aware of their HIV status, but this figure drops to 74% among those born in Southeast Asia [2]. Late‐ and advanced HIV diagnoses remain a significant challenge worldwide. This issue is particularly pronounced in some countries, such as China, Japan, Malawi, Zambia, Zimbabwe, and Lebanon, where over half of the new HIV diagnoses occurred at a late stage [5, 6, 7, 8]. A retrospective population‐level observational study of individuals diagnosed with HIV in Australia reported that the proportion of late HIV diagnoses was higher among the migrant population (45%) compared to all populations (37%) [2]. Additionally, factors associated with late diagnosis among migrants were individuals who reported male‐to‐male sex exposure, were born in countries with low HIV prevalence and came from non‐English speaking countries [9].

Timely HIV testing is essential for reducing the number of undiagnosed HIV cases, late diagnoses, and subsequent HIV‐related morbidity and mortality [10]. However, an estimated 24% of PLHIV globally have not tested for HIV, indicating existing barriers to HIV testing [1]. In Australia, laboratory‐based HIV testing is free if an individual has access to Medicare, the government‐funded universal healthcare insurance scheme. Those without Medicare (e.g., international students and workers on a working holiday visa) can also access free HIV testing through several channels, such as public sexual health services and nongovernmental organizations [11, 12]. A systematic review identified several barriers to HIV testing faced by migrants living in high‐income countries. Those barriers included cost, language barriers, lack of knowledge of HIV and the healthcare system in the host countries, low‐perceived risk of HIV, HIV‐related stigma and religious influences [13]. These barriers to HIV testing can lead to late diagnosis and, thus, delayed treatment.

Early diagnosis provides several benefits, including the reduction of HIV transmission from undiagnosed individuals and improved health outcomes through early treatment initiation [14, 15]. Understanding associated factors, facilitators and barriers to HIV testing among those with late‐ and advanced HIV diagnoses is essential to establish a strategy to improve early diagnoses. This study identified trends and factors associated with late (CD4 < 350 cells/µL) or advanced (CD4 < 200 cells/µL) HIV diagnoses at Melbourne Sexual Health Centre (MSHC), Australia. Additionally, we reported reasons associated with their HIV testing practice and risks of HIV infection reported among individuals with late‐ or advanced HIV diagnoses.

## 2 Methods

### 2.1 Data Collection and Setting

Our data were obtained from MSHC, Australia's largest public sexual health centre, which provides free testing and treatment for HIV and other sexually transmitted infections (STIs). MSHC conducted approximately 60 000 consultations and provided care for 1793 PLHIV in 2022 [16]. Upon registration, clients without prior HIV diagnosis completed a computer‐assisted self‐interviewing (CASI) collecting demographic characteristics, sexual behaviors and HIV testing history. HIV testing was offered to all clients. Clients with confirmed HIV received further tests, including CD4 cell count and HIV viral load.

Two researchers (A.T., C.K., M.M. or W.T.) independently reviewed electronic medical records, including consultation records, counseling notes and scanned documents, to extract data for each client with a new HIV diagnosis between 2011 and 2022. Extracted data included age, sex, gender, date of the last HIV test, date of HIV diagnosis, CD4 cell count and HIV viral load at the time of diagnosis, CD4 nadir, risk of HIV infections (e.g., condom use, number of sexual partners or overseas sex in the past 3 or 12 months before the HIV diagnosis), country of birth, year of arrival, Medicare status on the date of HIV diagnosis, STI diagnosis on the date of HIV diagnosis, history of STIs and antiretroviral therapy initiation, including name of therapy and date of initiation. Two researchers (A.T., C.K., M.M. or W.T.) independently reviewed consultation and counseling notes and categorized reasons associated with HIV testing practices and risks for contracting HIV, with discrepancies resolved by a third researcher. Ethics approval was obtained from the Alfred Hospital Ethics Committee, Melbourne, Australia (project 710/22). A consent waiver was obtained for this study since we used data obtained from routine clinical practice.

### 2.2 Study Populations, Subpopulations and Definitions

We included clients newly diagnosed with HIV at the MSHC or referred with new diagnoses from other healthcare facilities (e.g., primary care practices) between 2011 and 2022. Late diagnoses were defined as HIV diagnoses made when an individual's CD4 count was between 200 and 349 cells/µL and lower than 200 cells/µL for advanced diagnosis. Country income levels and regions were categorized based on The World Bank's criteria [17]. Due to the small number of clients from some regions, we classified geographical locations as Australia, “Latin America and the Caribbean,” “East Asia and the Pacific” and “Others.” We defined “new arrivals” as those arriving in Australia within 5 years of HIV diagnosis. “Medicare‐eligible” individuals included permanent residents, Australian or New Zealand citizens on the date of HIV diagnosis or those with Medicare recorded before or on the date of HIV diagnosis. We excluded duplicate records and individuals who (1) received an HIV diagnosis before 2011; (2) were not newly diagnosed (e.g., diagnosed overseas or self‐reported as being diagnosed previously elsewhere); and (3) lacked CD4 cell counts recorded within 6 months of their diagnosis. “Symptomatic individuals” were individuals who presented to the clinic with HIV‐ or STI‐related symptoms as a primary presenting complaint noted in triage reasons, CASI or consultation notes. “STI diagnoses on the date of HIV diagnosis” was co‐STI infections when first receiving HIV diagnosis. “STI screening” referred to asymptomatic testing, while “STI testing” referred to symptomatic cases. STIs included chlamydia, genital herpes or warts, gonorrhea, hepatitis B or C, *Mycoplasma genitalium*, nonspecific urethritis, syphilis and others. “Overseas sex” included having sex with someone outside or from outside Australia or New Zealand (e.g., had sex whilst traveling overseas or had sex in Australia with someone from overseas) in the past 12 months. ‘Reasons for testing for HIV when first diagnosed’ were the triage or other reasons that prompted individuals to visit the center to be tested for HIV. “Reasons for not testing for HIV earlier” were reported by clients during the counseling session. Examples of the reasons were: (1) LGBT‐related stigma, defined as avoiding HIV testing to prevent disclosing sexual orientation to family, friends or other people; (2) being a new arrival in Australia, defined as not knowing how to navigate the healthcare system as a new arrival; and (3) a low self‐perceived risk of HIV. “Risks for contracting HIV” were identified from counseling notes, where individuals speculated about their exposure.

### 2.3 Statistical Analysis

The proportion of late and advanced HIV diagnoses was calculated. The median and interquartile range (IQR) of age, CD4 cell counts and HIV viral load for each group were reported. Univariable and multivariable multinomial regression analyses were performed to identify the factors associated with late or advanced HIV diagnoses. Factors with *p* ≤ 0.2 in univariable regression analyses were considered potential confounding factors and were included in the multivariable regression analyses. We excluded variables with multicollinearity using variance inflation factors (VIF > 10) and correlation coefficient (> 0.5). We used backward stepwise regression to identify the final variables to include in the multivariable logistic regression analyses. Statistical significance was defined as *p* < 0.05. Crude and adjusted relative risk ratios (RRR) and the corresponding 95% confidence intervals (CIs) were reported. The chi‐square trend test was used to examine trends in the proportion of late‐ and advanced HIV diagnoses between 2011 and 2022. We examined the trends between 2011 and 2019 (before COVID‐19) as a sensitivity analysis. Additionally, we used descriptive statistics to report the risk of HIV infection, reasons for testing for HIV when first diagnosed and reasons for not testing for HIV earlier among those who had late‐ and advanced HIV diagnoses. All statistical analyses were performed using Stata (ver. 18.0; StataCorp LP, College Station, TX, USA).

## 3 Results

Among 656 new HIV diagnosis records, 50 were excluded due to duplicates (9), prior HIV diagnoses (5), and missing CD4 cell count recorded within 6 months of diagnosis (36). Among 606 individuals included in this analysis, 25% (152/606) had a late HIV diagnosis, 11% (65/606) had an advanced diagnosis, 98% (593/606) were male, and 94% (568/606) were men who had sex with men (MSM) (Table 1). Among individuals with late HIV diagnoses, the median age was 29 (IQR 26,38), 94% (143/152) were MSM, and 6% were heterosexuals (9/152). For advanced HIV diagnoses, the median age was 31 (IQR 26,42), 89% (58/65) were MSM, and 8% were heterosexuals (5/65) (Table 1).

**Table 1:** Demographic characteristics.

	All (%)	Late (%)	Advanced (%)
	606 (100)	152 (25)	65 (11)
Age (median, IQR, range)	30 (26,38, 18–71)	29 (IQR 25.5, 37.5; range 18–71)	31 (IQR 26, 42; range 18–71)
Sex and gender (*n*)			
Male	593 (98)	151 (99)	61 (94)
MSM	568 (94)	143 (94)	58 (89)
Heterosexual male	25 (4)	8 (5)	3 (5)
Heterosexual female	9 (1)	1 (1)	2 (3)
others	4 (1)	0 (0)	2 (3)
CD4 cell count (median, IQR, range, cell/µL)	430 (IQR 291,566; range 9–1679)	251 (IQR176,300; range 9–349)	130 (IQR 71,165; range 9–197)
Viral load (median, IQR, range)	27600 (IQR 6 217, 83700; range 19–10 000 000)	45200 (IQR 15 550, 123500; range 19–10 000 000)	68900 (IQR 32 300, 147000; range 73–3 210 000)
Overseas‐born (*n*)			
No	250 (41)	45 (30)	16 (25)
Yes	352 (58)	106 (70)	49 (75)
Unknown	4 (1)	1 (1)	0 (0)
Newly‐arrivals			
At least 5 years	129 (21)	34 (22)	13 (20)
Fewer than 5 years	207 (34)	68 (45)	33 (51)
Not reported	20 (3)	5 (3)	3 (5)
Not applicable (Australian‐born)	250 (41)	45 (30)	16 (25)
Region of birth			
Australia	250 (41)	45 (30)	16 (25)
Latin America and Caribbean	42 (7)	18 (12)	3 (5)
East Asia and Pacific	213 (35)	70 (46)	42 (65)
Others	101 (17)	19 (13)	4 (7)
Country income levels			
Low and middle	243 (40)	81 (53)	41 (63)
High	358 (102)	70 (66)	24 (49)
Not reported	5 (1)	1 (1)	0 (0)
Medicare eligibility on the date of diagnosis			
Yes	381 (63)	82 (54)	29 (45)
No	202 (33)	69 (45)	31 (48)
Unknown	23 (4)	1 (1)	5 (8)
Number of sexual partners in the past 3 months			
None or one	150 (25)	43 (28)	25 (38)
More than one	400 (66)	92 (61)	29 (45)
Missing	56 (9)	17 (11)	11 (17)
Number of sexual partners in the past 12 months			
None or one	74 (12)	24 (16)	14 (22)
More than one	486 (80)	116 (76)	46 (71)
Missing	46 (8)	12 (8)	5 (8)
Condom use in the past 3 months			
Not always	406 (67)	92 (61)	37 (57)
Always	111 (18)	35 (23)	6 (9)
Not reported^a^	89 (15)	21 (14)	13 (20)
Condom use in the past 12 months			
Not always	423 (70)	93 (61)	45 (69)
Always	123 (20)	42 (28)	7 (11)
Not reported^a^	60 (10)	16 (11)	10 (15)
HIV test in the past 12 months			
Yes	61 (10)	10 (7)	3 (5)
No	432 (71)	110 (72)	42 (65)
Not reported	113 (19)	32 (21)	20 (31)
STI diagnosis on the date of HIV diagnosis			
Yes	333 (55)	89 (59)	37 (57)
No	273 (45)	63 (41)	28 (43)
STI diagnosis in lifetime			
Yes	339 (56)	69 (45)	22 (34)
No	41 (27)	20 (31)	20 (31)
Not reported	42 (28)	23 (35)	23 (35)

Abbreviations: IQR, interquartile range; Medicare, the government‐funded universal healthcare insurance scheme; MSM, men who have sex with men; STI, sexually transmitted infection.

a No record of condom use (i.e., incomplete CASI or prefer not to answer).

### 3.1 Trends in the Proportion of Late and Advanced HIV Diagnoses Between 2011 and 2022

**Figure 1:**
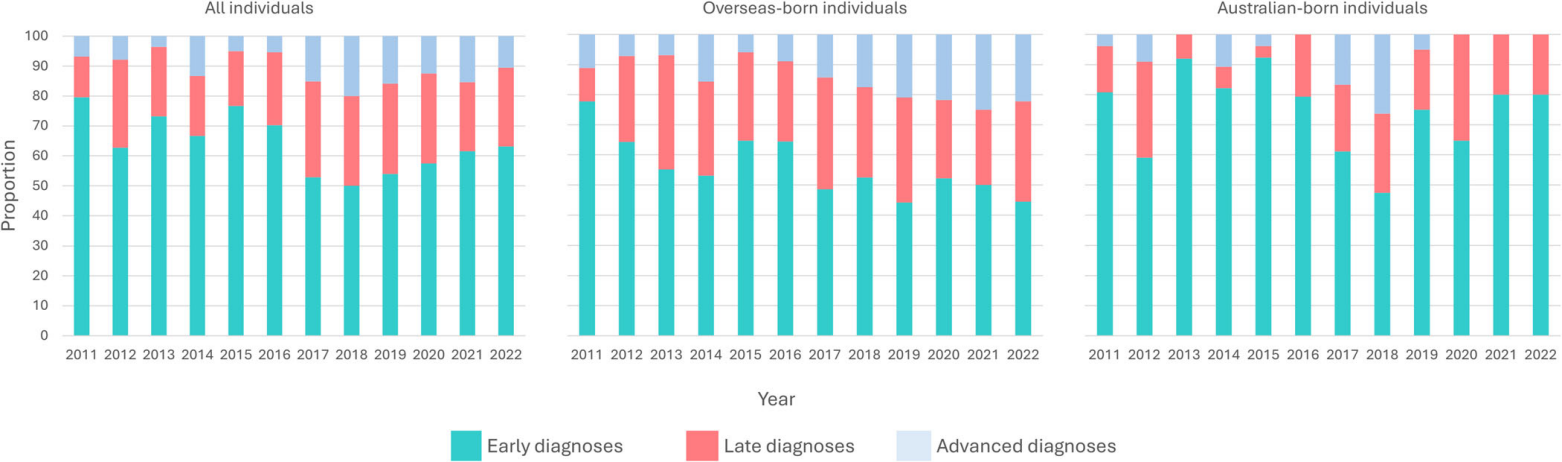
Percentage of delayed and advanced diagnoses among Australian‐ (*N* = 250) and overseas‐born individuals (*N* = 352).

Overall, the proportion of late or advanced HIV diagnoses increased from 21% (9/44) in 2011 to 37% (7/19) in 2022 (*P*_trend_ = 0.001). Specifically, the proportion of only advanced HIV diagnoses increased from 7% (3/44) in 2011 to 11% (2/19) in 2022 (*P*_trend_ = 0.011) (Figure 1). These findings did not change in the sensitivity analysis where the COVID‐19 years of 2020 to 2022 were removed (Supporting Information S1: Figure S1). Regarding HIV diagnoses among overseas‐born individuals, the proportion of late or advanced HIV diagnoses increased from 22% (4/18) in 2011 to 56% (5/9) in 2022 (*P*_trend_ = 0.017) and the proportion of only advanced diagnoses increased from 11% (2/18) in 2011 to 22% (2/9) in 2022 (*P*_trend_ = 0.008). In contrast, there was no significant change in the proportion of late or advanced diagnoses among Australian‐born (Figure 1 and Supporting Information S1: Table S1).

### 3.2 Factors Associated With Individuals With Late and Advanced HIV Diagnoses

**Figure 2:**
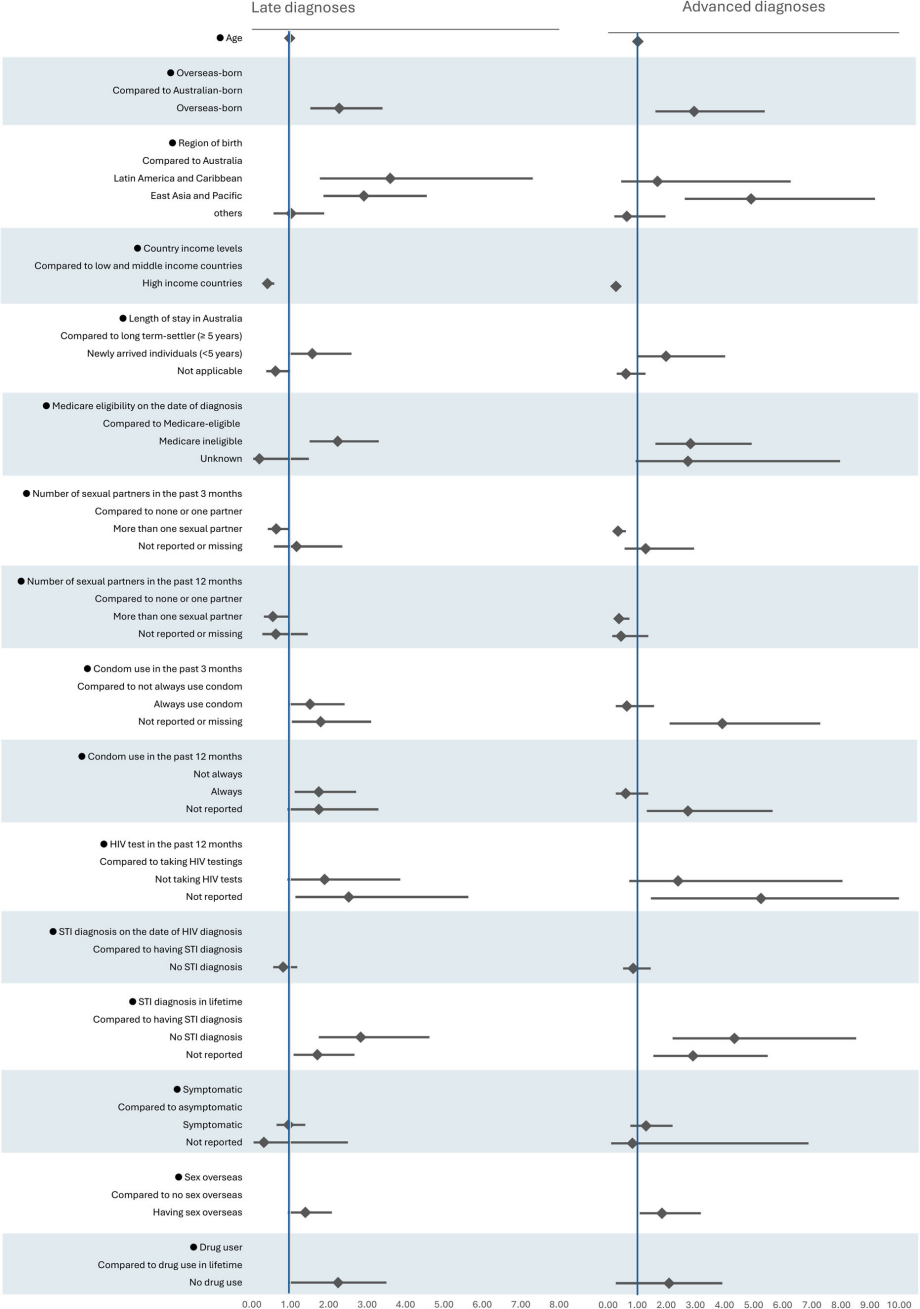
Relative importance of factors associated with late and advanced HIV diagnoses. RRR, relative risk ratio; STI, sexually transmitted infection.

In univariable multinomial regression analysis, compared to individuals born in Australia, those born in Latin America had a 3.60 times greater relative risk of a late HIV diagnosis (95% CI 1.77–7.31), while those born in East Asia and Pacific had a 2.91 times (1.86–4.55) greater relative risk of a late diagnosis and a 4.91 times (2.63–9.17) greater risk of an advanced diagnosis. Medicare‐ineligible individuals were at a 2.23 times higher risk of a late HIV diagnosis (RRR 2.23; 1.50–3.30) and an advanced diagnosis (RRR 2.83; 1.62–4.93). Compared to those who engaged in condomless sex in the last 12 months, individuals who did not have condomless sex had 1.74 times (1.11–2.71) greater relative risk of a late HIV diagnosis, and those who had no history of condom use had a 3.92 times (1.33–5.65) greater risk of an advanced HIV diagnosis. Individuals who had an STI diagnosis in their lifetime had a 2.38 times (1.74–4.62) greater relative risk of a late HIV diagnosis and a 4.34 times (2.21–8.52) higher risk of an advanced HIV diagnosis. Individuals who did not report drug use had a 2.24 times (1.44–3.50) greater relative risk of a late HIV diagnosis and a 2.09 times (1.12–3.92) higher risk of an advanced HIV diagnosis (Figure 2 and Supporting Information S1: Table S2).

**Figure 3:**
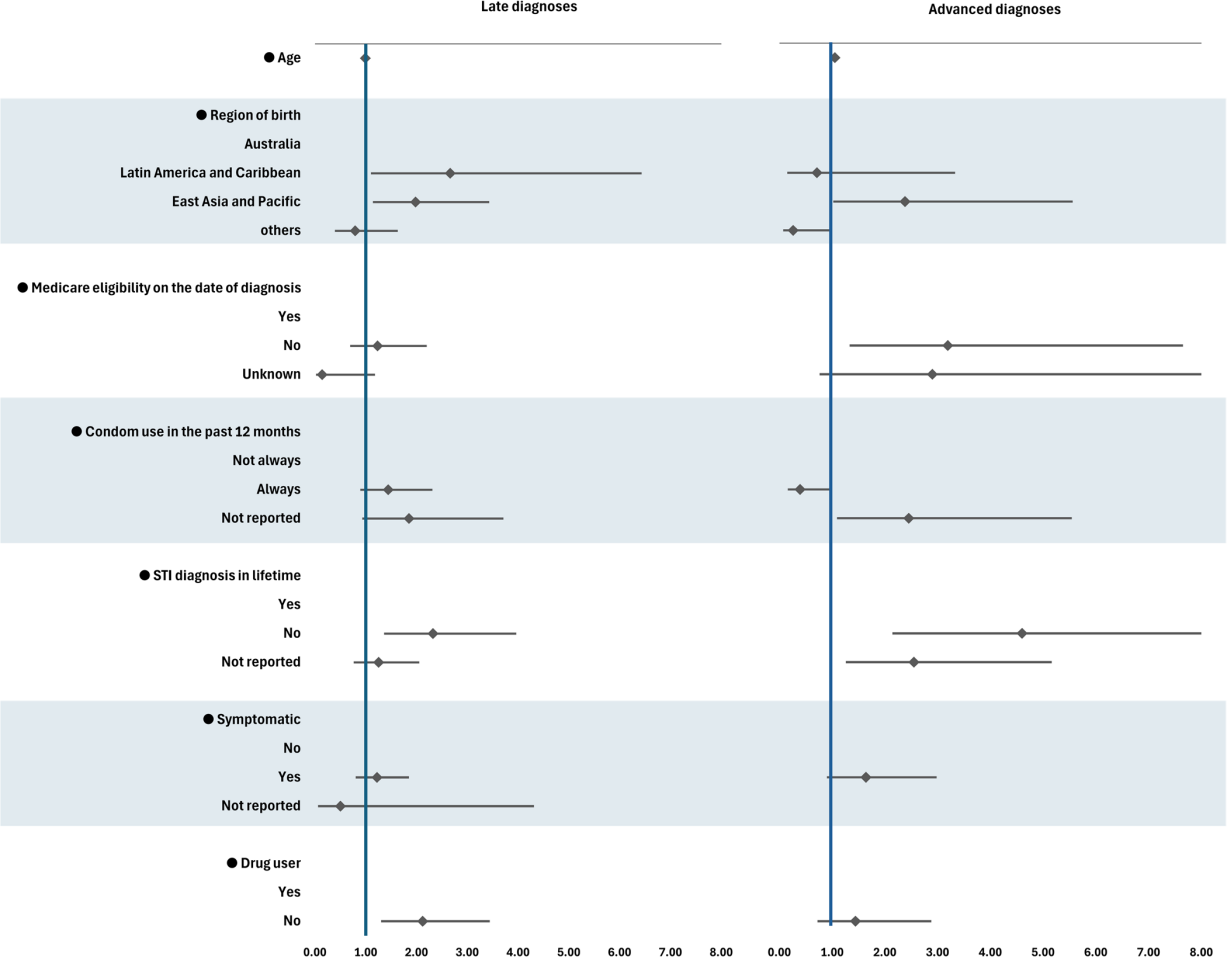
Adjusted relative importance of factors associated with late and advanced HIV diagnoses. RRR, relative risk ratio; STI, sexually transmitted infection.

In multivariable multinomial regression analysis, individuals who were born in Latin America and the Caribbean were at 2.66 times greater relative risk of a late diagnosis (adjusted relative risk ratio (aRRR) 2.66; 1.10–6.43), whereas those born in East Asia and Pacific were at 1.98 times (1.14–3.43) greater risk of a late diagnosis and at 2.38 times (1.02–5.56) higher risk of an advanced diagnosis. Individuals who did not have STI diagnoses in lifetime were at 2.32 times (1.36–3.96) higher risk of late diagnosis and at 4.60 times (2.14–9.92) higher risk of advanced diagnosis. Individuals who did not report drug use in lifetime had a 2.12 times (1.30–3.44) risk of late diagnosis. Individuals who were older (aRRR 1.05; 1.02–1.09), Medicare‐ineligible (aRRR 3.19; 1.33–7.65), had an unknown history of condom use in the last 12 months (aRRR 2.45; 1.09–5.54) more likely to have advanced HIV diagnosis (Figure 3 and Supporting Information S1: Table S2).

### 3.3 Reasons for Testing for HIV When First Diagnosed Among Individuals With Early, Late and Advanced HIV Diagnoses

The top three reasons for testing for HIV were STI screening (31%, 185/606), STI testing (25%, 154/606), and contact of an STI infection (10%, 59/606). Among individuals with late HIV diagnosis, the top reasons included STI testing (28%, 43/152), STI screening (26%, 39/152), and contact of a person with HIV (13%, 19/152). Among individuals with advanced HIV diagnosis, the top reasons included STI screening (32%, 21/65), STI testing (28%, 18/65) and contact of infection of HIV (14%, 9/65) (Supporting Information S1: Table S3). Additionally, among seven individuals who received a laboratory test following their positive HIV self‐test at home (HIVST was first available in 2019), one was diagnosed with late HIV and three with advanced HIV. All other reasons can be found in Supporting Information S1: Table S3.

### 3.4 Reasons for not Testing for HIV Earlier Among Individuals With Late‐ and Advanced HIV Diagnoses

The most common reasons for not testing for HIV earlier among individuals with late HIV diagnoses included being a new arrival in Australia (20%, 31/152), LGBT‐related stigma (20%, 30/152), and being in a committed relationship (11%, 17/152). Among those with advanced HIV diagnoses, the common reasons were being in a committed relationship (17%, 11/65), LGBT‐related stigma (17%, 11/65), being a new arrival in Australia (15%, 10/65), and having a low self‐perceived risk of HIV (11%, 7/65) (Supporting Information S1: Table S4).

### 3.5 Reported Risks for Contracting HIV Among Individuals With Late‐Stage and Advanced HIV Diagnoses

Individuals with new HIV diagnoses at late‐ and advanced stages reported similar risks of contracting HIV. Among 217 individuals with late or advanced HIV diagnoses, 55% (119/217) reported potential risks of their HIV infections. These risks included engaging in condomless anal sex with a casual partner (30%, 45/152 for late diagnoses and 54%, 35/65 for advanced diagnoses), participating in overseas sex (12%, 25/152 for late diagnosis and 12%, 8/65 for advanced diagnosis), and having sexual encounters with individuals living with HIV (8%, 12/152 for late diagnosis and 3%, 5/65 for advanced diagnosis) (Supporting Information S1: Table S5).

## 4 Discussion

Our study adds to the literature on the trends and factors associated with late and advanced HIV diagnoses, reasons for testing for HIV when first diagnosed, reasons for not testing earlier and risk of contracting HIV by analysing the data on new HIV diagnoses from the largest public sexual health center in Australia over the past decade. We found that individuals who were born overseas, particularly those from Latin America and the Caribbean, or East Asia and the Pacific; Medicare‐ineligible people; or individuals with a seemingly low risk of HIV infection (i.e., no STI diagnosis in lifetime, no drug use reported, and unknown history of condom use) had a greater risk of late HIV diagnoses. Being a new arrival in the country, being in a committed relationship and LGBT‐related stigma were factors associated with delayed testing for HIV among individuals with late and advanced HIV diagnoses. Innovative approaches to enhance accessibility to HIV testing are essential to increase early diagnoses.

Factors associated with late and advanced diagnoses vary by country setting. Cross‐sectional studies in Malawi, Zambia, and Zimbabwe identified older age and having sexual partners living with HIV [5], while a study in China identified male heterosexuals, people who inject drugs, and individuals with older age, low level of education and divorce were associated with late HIV diagnoses [6]. A retrospective review in Lebanon reported being older age was associated with late‐HIV diagnoses [7]. However, our findings did not show a significant association between late diagnoses and being male and drug use. This could be attributed to our study population, consisting primarily of males (98%). In previous reviews, migrants, particularly newly arrived international migrants, were found to be at increased risk of HIV [18, 19]. Inequitable access and a lack of knowledge to navigate HIV services in the host countries could dissuade migrants from accessing HIV services [20]. For instance, in Australia, individuals with Medicare have broad access to free HIV testing, whereas those without Medicare are typically restricted to accessing free testing only through specific facilities, such as publicly funded sexual health clinics [21]. Despite these barriers, all people living with HIV in Australia—regardless of Medicare status—are eligible to receive free antiretroviral therapy (ART) [22]. Additionally, an observational study reported that migrants born in Southeast Asia and sub‐Saharan Africa were associated with late diagnoses between 2008 and 2017 in Australia [9]. Meanwhile, our study found that individuals born in Latin America and East Asia‐Pacific, including Southeast Asia, were associated with late diagnoses. These findings emphasize the need for up‐to‐date tailored approaches to increase HIV testing and reach specific populations in different countries.

Seemingly low risk of HIV infection does not equate to no risk. Our study uncovered individuals with a self‐identified low risk of HIV infection, including no prior STI diagnosis, no prior drug use, or an unknown history of condom use, were associated with late‐stage diagnoses, and those with an advanced HIV diagnosis reported low‐self‐perceived risk. Similarly, a qualitative study in the Netherlands revealed that low‐risk perception hindered timely HIV testing among those diagnosed at a late stage [23]. In Australia, HIV testing is recommended for individuals at substantial risk of HIV infection, such as people who inject drugs or those who have condomless anal sex [24]. In our study, over half of participants were diagnosed when receiving HIV screening as recommended. However, while one in ten MSM was diagnosed with advanced HIV, one in four heterosexuals was diagnosed with advanced HIV. Although heterosexuals are typically considered to be at low risk of HIV, there has been a declining trend in HIV testing within this group [25]. Therefore, the greater odds of advanced HIV diagnoses among heterosexuals may be attributed to lower HIV testing rates in this population. Additionally, those with an unknown history of condom use were more likely to have a late diagnosis. This result aligned with the previous cross‐sectional study from our center in which people who declined to report their sexual practices on CASI were more likely to have STIs [26]. Together, these data suggest we must change the paradigm of who clinicians may offer HIV testing to, that is, not to rely on risk‐based screening.

Innovative approaches to overcome the specific barriers faced by underserved populations are essential.HIV self‐tests were proven to increase testing rates without negatively affecting linkage to HIV and STI care or causing social harm [27, 28]. online HIV‐self testing effectively engaged suboptimal testers, including international migrants [29, 30, 31]. The Australian Government has expanded free self‐testing kit availability from 3000 in 2023 to 14 000 in 2024 [32]. Free kits can be accessed via HIVtest.au or vending machines or purchased online or at pharmacies for 20–30 Australian dollars. At the healthcare level, healthcare professionals should consider more inclusive HIV risk assessment methods, moving beyond traditional risk checklists to effectively identify individuals with undiagnosed HIV. For example, opt‐out testing policies can normalize HIV testing and ensure that it is a routine part of medical care rather than specifically targeting individuals [33]. This approach could increase the testing rate among underserved populations, such as heterosexuals, migrants and individuals in a committed relationship. Opt‐out HIV testing is recommended as part of comprehensive STI testing in Australia [34]. Additionally, a shop‐front rapid point‐of‐care HIV testing service run by the community can reach people who are never tested [35]. Social network‐based approaches [36] or behavioral economic approaches using “Nudges” [37, 38] could increase the uptake of HIV testing and should be adapted and implemented in underserved populations. Collectively, these efforts can contribute to earlier diagnoses, better health outcomes and reduced HIV transmission within the community.

The study's strength was a decade of detailed clinical data on new HIV diagnoses from the largest sexual health center in Australia. There were several limitations. First, as a retrospective study, some variables, such as details of sexual orientation, chemsex and country of birth of sexual partners, might be missing. However, we manually reviewed each client record, and our centre collected detailed demographic characteristics, sexual behaviors, and counseling notes, enabling a comprehensive analysis of each diagnosis. Second, our study period covered the COVID‐19 pandemic year (i.e., 2020), during which individuals reported fewer sexual partners and reduced clinic attendances, potentially contributing to the higher odds of late HIV diagnoses as asymptomatic individuals were less likely to obtain HIV testing during this period [39]. Additionally, the border closure contributed to the reduced number of overseas‐born individuals. However, our sensitivity analysis, limiting the trend test up to 2019, showed similar findings. Third, the reported risks for contracting HIV and reasons for not testing earlier were inferred from the counseling notes, which we could report only data available in the counseling notes. However, this approach allowed us to analyse a large number of individuals' reports, which would be challenging in a qualitative study. Fourth, the study participants were from a single centre in the urban area of Melbourne, leading to potential selection bias, and our findings may not be generalizable to people newly diagnosed outside of Melbourne, Australia. However, new HIV cases in this area accounted for 88% (194/220) of new HIV cases in Victoria and 27% (194/722) in Australia in 2023 [4, 40]. Fifth, changes in immigration patterns during the study period may have influenced the observed increase in late and advanced HIV diagnoses among overseas‐born individuals. While the proportion of Australian residents born in East Asia and the Pacific increased between 2011 and 2022, the proportion born in Latin America remained stable [41]. Although the ‘newly arrivals’ variable was initially included in our multivariable models to account for immigration effects, it was removed due to multicollinearity with the ‘region of birth’ variable. Consequently, we were unable to fully disentangle the effects of immigration trends from regions of birth. This potential confounding should be considered when interpreting the observed associations.

## 5 Conclusions

The proportions of late and advanced HIV diagnoses have increased over a decade, particularly among overseas‐born individuals. Individuals who were Medicare‐ineligible and those with a seemingly low risk of HIV infection were at greater risk of late or advanced HIV diagnoses. A recent arrival in the country, being in a committed relationship and LGBT‐related stigma were reported as reasons for not testing earlier. Broadening the scope of HIV testing can lead to earlier diagnoses and a reduction in overall transmission rates.

## Author Contributions

Jason J. Ong and Warittha Tieosapjaroen conceived the idea. Arron Tran, Cham‐mill Kim, Marcus Maisano, and Warittha Tieosapjaroen reviewed and extracted data from the electronic medical records and categorized the reasons associated with HIV testing practices and risks for contracting HIV. Arron Tran, Cham‐mill Kim, Marcus Maisano, Warittha Tieosapjaroen, and Jason J. Ong wrote the first draft of the manuscript. Eric P. F. Chow provided statistical advice. Warittha Tieosapjaroen revised and finalized the manuscript. All authors contributed to the manuscript and approved the final version for submission.

## Ethics Statement

Ethics approval was obtained from the Alfred Hospital Ethics Committee, Melbourne, Australia (project 710/22). A consent waiver was obtained for this study since we used data obtained from routine clinical practice.

## Conflicts of Interest

The authors declare no conflicts of interest.

## Supporting information

Supplementary late HIV diagnosis V2.

## Data Availability

The data are not publicly available due to their containing information that could compromise the privacy of research participants.
