# Supplementary material for: Trend and Associated Factors for Late and Advanced HIV Diagnoses in 2011–2022 in Melbourne, Australia
Source: J Med Virol. 2025 Jun 4;97(6):e70430. doi: 10.1002/jmv.70430 (PMC12135748; doi:10.1002/jmv.70430)
Supplement: Supplementary file 1 — Supplementary late HIV diagnosis V2. [file JMV-97-e70430-s001.docx]

**Figure S1. Trends of late and advanced HIV diagnoses at Melbourne Sexual Health Centre between 2011-2019**

**Table S1. Number of early, late and advanced HIV diagnoses between 2011 and 2022**

| **Year** | **Total number of HIV diagnoes** | **Number of early diagnoses** | **Number of late HIV diagnoses** | **Number of advanced HIV diagnoses** |
| --- | --- | --- | --- | --- |
| 2011 | 44 | 35 | 6 | 3 |
| 2012 | 51 | 32 | 15 | 4 |
| 2013 | 56 | 41 | 13 | 2 |
| 2014 | 60 | 40 | 12 | 8 |
| 2015 | 60 | 46 | 11 | 3 |
| 2016 | 74 | 52 | 18 | 4 |
| 2017 | 53 | 28 | 17 | 8 |
| 2018 | 60 | 30 | 18 | 12 |
| 2019 | 63 | 34 | 19 | 10 |
| 2020 | 40 | 23 | 12 | 5 |
| 2021 | 26 | 16 | 6 | 4 |
| 2022 | 19 | 12 | 5 | 2 |

**Table S2.** **Factors associated with individuals with late and advanced HIV diagnoses**

|  | **Late diagnoses** | | | | | | | **Advanced diagnoses** | | | | | | |
| --- | --- | --- | --- | --- | --- | --- | --- | --- | --- | --- | --- | --- | --- | --- |
|  | **RRR** | **95% CI** | **(N=65)** | **aRRR** | 95%CI | **P-value** | **RRR** | | **95% CI** | **P-value** | **aRRR** | **95% CI** | **P-value** |  |
| **Age** | 0.98 | (0.96 to 1) | 0.102 | 0.99 |  | 0.672 | 1.02 | | (0.99 to 1.04) | 0.223 | 1.05 | (1.02 to 1.09) | 0.003 |  |
| **Overseas-born** |  |  |  |  |  |  |  | |  |  |  |  |  |  |
| No | ref |  |  |  |  |  | ref | |  |  |  |  |  |  |
| Yes | 2.27 | (1.52 to 3.4) | <0.001 |  |  |  | 2.95 | | (1.62 to 5.38) | <0.001 |  |  |  |  |
| Unknown | 1.05 | (0.11 to 9.62) | 0.965 |  |  |  | Omitted | |  |  |  |  |  |  |
| **Region of birth** |  |  |  |  |  |  |  | |  |  |  |  |  |  |
| Australia | ref |  |  | ref |  |  | ref | |  |  | ref |  |  |  |
| Latin America and the Caribbean | 3.60 | (1.77 to 7.31) | <0.001 | 2.66 | (1.1 to 6.43) | 0.030 | 1.69 | | (0.45 to 6.27) | 0.435 | 0.71 | (0.15 to 3.33) | 0.661 |  |
| East Asia and the Pacific | 2.91 | (1.86 to 4.55) | <0.001 | 1.98 | (1.14 to 3.43) | 0.016 | 4.91 | | (2.63 to 9.17) | <0.001 | 2.38 | (1.02 to 5.56) | 0.045 |  |
| others | 1.02 | (0.56 to 1.88) | 0.945 | 0.79 | (0.39 to 1.63) | 0.527 | 0.64 | | (0.21 to 1.97) | 0.436 | 0.26 | (0.07 to 0.95) | 0.042 |  |
| Not reported | Omitted |  |  | omitted |  |  | Omitted | |  |  | Omitted |  |  |  |
| **Country income levels** |  |  |  |  |  |  |  | |  |  |  |  |  |  |
| Low and Middle | ref |  |  |  |  |  | ref | |  |  |  |  |  |  |
| High | 0.40 | (0.27 to 0.58) | <0.001 |  |  |  | 0.27 | | (0.16 to 0.46) | <0.001 |  |  |  |  |
| Not reported | 0.37 | (0.04 to 3.4) | 0.382 |  |  |  | Omitted | |  |  |  |  |  |  |
| **Newly-arrival** |  |  |  |  |  |  |  | |  |  |  |  |  |  |
| At least 5 years | ref |  |  |  |  |  | ref | |  |  |  |  |  |  |
| Fewer than 5 years | 1.57 | (0.95 to 2.59) | 0.080 |  |  |  | 1.99 | | (0.98 to 4.02) | 0.056 |  |  |  |  |
| Not reported | 0.61 | (0.37 to 1.01) | 0.055 |  |  |  | 0.61 | | (0.29 to 1.28) | 0.192 |  |  |  |  |
| **Medicare eligibility on the date of diagnosis** |  |  |  |  |  |  |  | |  |  |  |  |  |  |
| Yes | ref |  |  | ref |  |  | ref | |  |  | ref |  |  |  |
| No | 2.23 | (1.5 to 3.3) | <0.001 | 1.23 | (0.69 to 2.2) | 0.475 | 2.83 | | (1.62 to 4.93) | <0.001 | 3.19 | (1.33 to 7.65) | 0.01 |  |
| Unknown | 0.19 | (0.03 to 1.48) | 0.113 | 0.14 | (0.02 to 1.18) | 0.071 | 2.74 | | (0.94 to 7.97) | 0.065 | 2.90 | (0.76 to 11.09) | 0.120 |  |
| **Number of sexual partners in the past 3 months** |  |  |  |  |  |  |  | |  |  |  |  |  |  |
| None or one | ref |  |  |  |  |  | ref | |  |  |  |  |  |  |
| More than one | 0.63 | (0.41 to 0.97) | 0.038 |  |  |  | 0.34 | | (0.19 to 0.61) | <0.001 |  |  |  |  |
| Not reported | 1.16 | (0.57 to 2.35) | 0.684 |  |  |  | 1.29 | | (0.56 to 2.95) | 0.549 |  |  |  |  |
| **Number of sexual partners in the past 12 months** |  |  |  |  |  |  |  | |  |  |  |  |  |  |
| One and lower | ref |  |  |  |  |  | ref | |  |  |  |  |  |  |
| More than one | 0.54 | (0.31 to 0.94) | 0.029 |  |  |  | 0.37 | | (0.18 to 0.73) | 0.004 |  |  |  |  |
| Not reported | 0.62 | (0.27 to 1.45) | 0.270 |  |  |  | 0.44 | | (0.14 to 1.38) | 0.159 |  |  |  |  |
| **Condom use in the past 3 months** |  |  |  |  |  |  |  | |  |  |  |  |  |  |
| Not always | ref |  |  |  |  |  | ref | |  |  |  |  |  |  |
| Always | 1.51 | (0.94 to 2.41) | 0.088 |  |  |  | 0.64 | | (0.26 to 1.58) | 0.335 |  |  |  |  |
| Not reported | 1.79 | (1.04 to 3.1) | 0.037 |  |  |  | 3.92 | | (2.11 to 7.29) | <0.001 |  |  |  |  |
| **Condom use in the past 12 months** |  |  |  |  |  |  |  | |  |  |  |  |  |  |
| Not always | ref |  |  | ref |  |  | ref | |  |  | ref |  |  |  |
| Always | 1.74 | (1.11 to 2.71) | 0.015 | 1.44 | (0.89 to 2.31) | 0.138 | 0.60 | | (0.26 to 1.38) | 0.230 | 0.39 | (0.16 to 0.96) | 0.040 |  |
| Not reported | 1.74 | (0.92 to 3.29) | 0.091 | 1.85 | (0.93 to 3.71) | 0.082 | 2.74 | | (1.33 to 5.65) | 0.006 | 2.45 | (1.09 to 5.54) | 0.031 |  |
| **HIV test in the past 12 months** |  |  |  |  |  |  |  | |  |  |  |  |  |  |
| Yes | ref |  |  |  |  |  | ref | |  |  |  |  |  |  |
| No | 1.89 | (0.92 to 3.86) | 0.083 |  |  |  | 2.40 | | (0.72 to 8.05) | 0.156 |  |  |  |  |
| Not reported | 2.52 | (1.13 to 5.63) | 0.024 |  |  |  | 5.25 | | (1.47 to 18.9) | 0.011 |  |  |  |  |
| **STI diagnosis on the date of HIV diagnosis** |  |  |  |  |  |  |  | |  |  |  |  |  |  |
| Yes | ref |  |  |  |  |  | ref | |  |  |  |  |  |  |
| No | 0.81 | (0.55 to 1.18) | 0.263 |  |  |  | 0.86 | | (0.51 to 1.46) | 0.579 |  |  |  |  |
| **STI diagnosis in lifetime** |  |  |  |  |  |  |  | |  |  |  |  |  |  |
| Yes | ref |  |  | ref |  |  | ref | |  |  | ref |  |  |  |
| No | 2.83 | (1.74 to 4.62) | <0.001 | 2.32 | (1.36 to 3.96) | 0.002 | 4.34 | | (2.21 to 8.52) | <0.001 | 4.60 | (2.14 to 9.92) | <0.001 |  |
| Not reported | 1.70 | (1.08 to 2.67) | 0.022 | 1.25 | (0.76 to 2.05) | 0.372 | 2.91 | | (1.55 to 5.48) | 0.001 | 2.55 | (1.26 to 5.16) | 0.009 |  |
| **Symptomatic** |  |  |  |  |  |  |  | |  |  |  |  |  |  |
| No | ref |  |  | ref |  |  | ref | |  |  | ref |  |  |  |
| Yes | 0.95 | (0.64 to 1.39) | 0.775 | 1.22 | (0.8 to 1.85) | 0.352 | 1.30 | | (0.76 to 2.21) | 0.333 | 1.64 | (0.9 to 2.98) | 0.105 |  |
| Not reported | 0.31 | (0.04 to 2.5) | 0.271 | 0.50 | (0.06 to 4.31) | 0.529 | 0.83 | | (0.1 to 6.88) | 0.867 | omitted |  |  |  |
| **Overseas sex** |  |  |  |  |  |  |  | |  |  |  |  |  |  |
| No | ref |  |  |  |  |  | ref | |  |  |  |  |  |  |
| Yes | 1.39 | (0.933 to 2.08) | 0.105 |  |  |  | 1.85 | | (1.08 to 3.18) | 0.026 |  |  |  |  |
| **Drug user in lifetime** |  |  |  |  |  |  |  | |  |  |  |  |  |  |
| Yes | ref |  |  | ref |  |  | ref | |  |  | ref |  |  |  |
| No | 2.24 | (1.44 to 3.5) | <0.001 | 2.12 | (1.3 to 3.44) | 0.002 | 2.09 | | (1.12 to 3.92) | 0.021 | 1.44 | (0.72 to 2.88) | 0.302 |  |

CI=confidence interval, ref=reference level, RRR=relative risk ratio, aRRR=adjusted relative risk ratio, STI= sexually transmitted infection

**Table S3. Reason for testing for HIV when first diagnosed among individuals with early, late and advanced HIV diagnoses**

|  | **Early diagnoses** | **Late diagnoses** | **Advanced diagnoses** | **Total**  **(N=606)** |
| --- | --- | --- | --- | --- |
|  | **(N = 389)** | **(N=152)** | **(N=65)** |  |
|  | **n (%)** | **n (%)** | **n (%)** | **n (%)** |
| STI screening | 125 (32) | 39 (26) | 21 (32) | 185 (31) |
| STI testing | 93 (24) | 43 (28) | 18 (28) | 154 (25) |
| Contact of infection of an STI | 38 (10) | 14 (9) | 7 (11) | 59 (10) |
| Contact of infection of HIV | 26 (7) | 19 (13) | 9 (14) | 54 (9) |
| Engaging in unexpected condomless sex or request or eligible for PEP | 19 (5) | 3 (2) | 1 (2) | 22 (4) |
| Visa requirement | 3 (1) | 0 (0) | 0 (0) | 3 (0) |
| Sexual health certificate for sex workers | 2 (1) | 1 (1) | 0 (0) | 3 (0) |
| Positive HIV self-testing | 4 (1) | 1 (1) | 2 (3) | 7 (1) |
| Requesting PrEP | 3 (1) | 0 (0) | 0 (0) | 3 (0) |
| Other | 5 (1) | 3 (2) | 2 (3) | 10 (2) |
| Not reported | 71 (18) | 29 (19) | 5 (8) | 106 (17) |

STI= sexually transmitted infection, PEP=post-exposure prophylaxis, PrEP=Pre-exposure prophylaxis

*STI tests were conducted when clients reported no STI-related symptoms

^†^STI tests were conducted when clients reported STI-related symptoms

**Table S4. Reasons for not testing for HIV earlier among individuals with late (N=152) and advanced HIV (N=65) diagnoses**

|  | **Late diagnoses** | **Advanced diagnoses** |
| --- | --- | --- |
|  | **n (%)** | **n (%)** |
| Committed relationship | 17 (11) | 11 (17) |
| Always using condoms | 13 (9) | 2 (3) |
| Believing that their sexual partners were on PrEP or PEP | 6 (4) | 1 (2) |
| LGBT-related stigma | 30 (20) | 11 (17) |
| HIV-related stigma | 9 (6) | 4 (6) |
| Recent HIV testing | 7 (5) | 3 (5) |
| New to Australia | 31 (20) | 10 (15) |
| Concern about confidentiality when testing for HIV | 5 (3) | 1 (2) |
| No symptoms | 1 (1) | 1 (2) |
| Low-risk perception of HIV | 7 (5) | 7 (11) |
| Others | 8 (5) | 6 (9) |

LGBT=Lesbian, gay, bisexual, transgerder, PEP=Post-exposure prophylaxis, PrEP=Pre-exposure prophylaxis, STI=sexually transmitted infection

**Table S5. Reported risks of HIV infections**

|  | **Late diagnoses** | **Advanced diagnoses** |
| --- | --- | --- |
|  | **n (%)** | **n (%)** |
| Condomless anal sex | 45 (30) | 20 (13) |
| Overseas sex | 17 (11) | 8 (5) |
| Sexual partners living with HIV | 12 (8) | 5 (3) |
| Chemsex | 5 (3) | 0 (0) |
| Group sex | 3 (2) | 0 (0) |
| Paying for commercial sex | 2 (1) | 2 (1) |
| Ceasing PrEP or PEP | 0 (0) | 0 (0) |

PEP=Post-exposure prophylaxis, PrEP=Pre-exposure prophylaxis
